# Standardized reporting of pre-analytical variables and quality control of plasma and serum to enhance rigor and reproducibility in liquid biopsy research

**DOI:** 10.1016/j.jlb.2024.100163

**Published:** 2024-07-25

**Authors:** Fabrice Lucien, Dakota Gustafson

**Affiliations:** Department of Urology, Mayo Clinic, Rochester, MN, USA; Department of Immunology, Mayo Clinic, Rochester, MN, USA; Department of Laboratory Medicine & Pathobiology, University of Toronto, Toronto, Ontario, Canada; Toronto General Hospital Research Institute, Toronto, Ontario, Canada

Extracellular vesicles (EVs) have emerged as pivotal components in the field of liquid biopsy, reflecting the molecular cargo of their cells of origin. Their potential in diagnostics, especially in oncology, is profound. However, the reproducibility of EV-based biomarker studies remains a significant challenge due to pre-analytical variability. To address this, The Blood Task Force of the International Society for Extracellular vesicles (ISEV) recently published the MIBlood-EV: Minimal Information to Enhance the Quality and Reproducibility of Blood Extracellular Vesicle Research,” [1].

The MIBlood-EV is a comprehensive tool designed to record and report pre-analytical variables and quality control measures for plasma and serum samples (**Supplementary File**). It includes 27 items across three sections: general study information, blood collection/processing/storage procedures, and plasma/serum quality control. The framework specifically addresses common confounders in EV preparations, such as hemolysis, residual platelets, and lipoproteins, which can significantly impact the integrity and analysis of EV samples. The MIBlood-EV allows researchers to report the quality of plasma and serum samples qualitatively (e.g visual examination of hemolysis) and quantitatively using standard methodological guidelines (e.g haematology analyzer for platelet count). Researchers are invited to include the completed MIBlood-EV report as a supplementary file in published manuscripts and to upload it in a shared data repository [1]. Systematic analysis of this real-world information will help develop evidence-based guidelines and expert consensus statements for standardized EV-dedicated research protocols and reporting practices in the field.

Our initiative responds to the scientific community's call for improved rigor and transparency in EV research. By providing detailed reporting guidelines, the MIBlood-EV framework aims to enhance the consistency and reproducibility of studies, facilitating more reliable conclusions and advancing the field of liquid biopsy. We believe this tool will be instrumental for researchers and biobanks in standardizing their protocols and improving the quality of stored samples.

Importantly, the principles outlined in the MIBlood-EV can be broadly applied to other liquid biopsy biomarkers, such as circulating tumor cells (CTCs) and cell-free DNA (cfDNA). CTCs and cfDNA are integral components of liquid biopsy but these biomarkers also suffer from significant pre-analytical variability, which can affect their analysis and interpretation [2]. For instance, the viability of CTCs can be compromised by delays in processing or suboptimal storage conditions, and cfDNA can degrade rapidly if not properly handled. Such variability can lead to inconsistent results and hinder the clinical implementation of these biomarkers. Few studies have benchmarked pre-analytical procedures for cfDNA and provided evidence-based recommendations for the development of standardized protocols [3,4]. By adopting the MIBlood-EV framework, researchers working with CTCs and cfDNA can benefit from a structured approach to reporting pre-analytical variables and quality of prepared samples for downstream analysis. Practically, we could envision the creation of a standardization working group within the International Society of Liquid Biopsy (ISLB) whose objective would be to generate a “minimum information standard” consisting of pre-analytical guidelines and a common format to report data (e.g MIBlood-DNA, MIBlood-CTC). This would not only enhance the quality and reproducibility of liquid biopsy studies but also facilitate inter-laboratory comparative studies using different assays. Finally, it would improve the quality of banked biospecimens shared between biorepositories and research laboratories.

We invite the liquid biopsy research community to adopt the MIBlood-EV framework and integrate it into their EV studies. By doing so, we can collectively advance the quality and reproducibility of EV research, enhancing its clinical utility and ultimately advance the field of precision medicine.

## Declaration of competing interest

The authors declare the following financial interests/personal relationships which may be considered as potential competing interests:Fabrice Lucien reports a relationship with Mayo Clinic Minnesota that includes: consulting or advisory, equity or stocks, and funding grants. If there are other authors, they declare that they have no known competing financial interests or personal relationships that could have appeared to influence the work reported in this paper.
