# Supplementary material for: Standardized reporting of pre-analytical variables and quality control of plasma and serum to enhance rigor and reproducibility in liquid biopsy research
Source: J Liq Biopsy. 2024 Jul 25;6:100163. doi: 10.1016/j.jlb.2024.100163 (PMC11863886; doi:10.1016/j.jlb.2024.100163)
Supplement: Multimedia component 1 [file mmc1.pdf]

INTERNATIONAL SOCIETY for EXTRACELLULAR VESICLES

MIBlood-EV

Standardized Reporting Tool for Blood EV Research (Human)

STUDY INFORMATION

1.0 Manuscript title			
1.1 Corresponding author (Name and Email)			
1.2 Institution name			
1.3 Time period of experiment (e.g. 2022-2024)		1.4 Number of samples	
1.5 Cargo of interest	☐ Vesicles ☐ Protein ☐ RNA ☐ DNA ☐ Other:		
1.6 Biospecimen type	☐ Plasma ☐ Serum	1.7 Biospecimen state	
1.8 Source of frozen specimens		1.9 Years of collection (range)	

BLOOD COLLECTION AND PROCESSING

2.0 Patient fasting status		2.1 Fasting length (e.g. hours/days)	
2.2 Anatomical access site		2.3 Needle diameter (e.g. gauge)	
2.4 Blood volume collected (mL)			
2.5 Plasma anticoagulant	☐ EDTA ☐ Citrate ☐ Heparin ☐ Other:		
2.6 Serum tube type		2.7 Serum clotting time (minutes)	
2.8 Time between collection and first centrifugation (range in hours)			
2.9 Transport temperature		2.10 Transport condition of tubes	
2.11 Centrifuge brand and model			
2.12 Bucket rotor type		2.13 Number of centrifugation cycles	
2.14a 1st Centrifugation speed (RCF in x g)		2.14b 1st Centrifugation time (minutes)	
2.15 1st Rotor brake		2.16 1st Centrifugation temperature	
2.17a 2nd Centrifugation speed (RCF in x g)		2.17b 2nd Centrifugation time (minutes)	
2.18 2nd Rotor brake		2.19 2nd Centrifugation temperature	
2.20 Additional processing steps (e.g. filtration)			
2.21 Storage tubes (brand, type, source, catalog number)			
2.22 Storage temperature		2.23 Length of storage (range in years)	

PLASMA/SERUM QUALITY CONTROL

3.0 Number of freeze-thaw cycles (range)			
3.1 Thawing temperature		3.2 Thawing duration (minutes)	

Hemolysis

3.3 Presence of hemolysis		3.4 Frequency of hemolyzed samples (e.g. <25%, 25-50%)	
3.5 Method used		3.6 RBC count (Median, 95% CI, N)	
3.7 RBC counter brand and type			
3.8 Spectrophotometry hemoglobin concentration (mean g/L)			
3.9 Spectrophotometer brand, model and wavelength measured (e.g. 414 nm)			
3.10 Hemolized samples were discarded			

Platelets

3.11 Presence of platelets		3.12 Method used (e.g. Flow Cytometry)	
3.13 Marker(s) used (e.g. CD61, CD41)			
3.14 Concentration (median, 95% CI, N)			
3.15 Platelet counter instrument brand, type and limit of detection (cells/L)			
3.16 Flow cytometer brand and type			
3.17 Flow cytometry size and fluorescence ranges of detection in nanometers and MESF, respectively			

Lipoproteins

3.18 Presence of lipoproteins		3.19 Method used (WB, ELISA, FC)	
3.20 Spectrophotometry L-index			
3.21 Spectrophotometer brand, model and wavelength measured (e.g. 700 nm)			
3.22 WB Marker(s) used (e.g. Apo B)			
3.23 Western blot images provided in manuscript?			
3.24 Flow cytometry marker(s) used (e.g. ApoB)			
3.25 Flow cytometry concentration (median, 95% CI, N)			
3.26 Flow cytometer brand and type			
3.27 Flow cytometry size and fluorescence ranges of detection in nanometers and MESF, respectively			
3.28 Brand and catalog number for ELISA kit used for each Apolipoprotein test (e.g. ApoB)			
3.29 Concentration measured for each Apolipoprotein tested (mean µg/mL)			
